# Downregulation of hsa_circ_0004543 Activates oxLDL-Induced Vascular Endothelial Cell Proliferation and Angiogenesis

**DOI:** 10.3389/fgene.2021.632164

**Published:** 2021-03-24

**Authors:** Lu Han, Dandan Li, Yanwen Hang, Xiaojuan Zong, Jiashun Lv, Xiaolu Bai, Yi Lu, Peng Zhang, Meiling Zhou, Zhaodi Wu, Wei Hu

**Affiliations:** Department of Cardiology, Minhang Hospital, Fudan University, Shanghai, China

**Keywords:** hsa_circ_0004543, atherosclerosis, angiogenesis, vascular endothelial cells, treatment

## Abstract

Circular RNAs (circRNAs) are novel non-coding RNAs, which show abnormal expression in several diseases, such as atherosclerosis (AS). The purpose of the present study was to reveal the association between hsa_circ_0004543 and AS. In the present study, hsa_circ_0004543 was overexpressed in human umbilical vein endothelial cells (HUVECs) induced by oxidized low-density lipoprotein (oxLDL). Inhibition of hsa_circ_0004543 expression facilitated the proliferation, migration, and invasion of HUVECs and significantly reduced their apoptotic rate following treatment with oxLDL. Furthermore, silencing of hsa_circ_0004543 activated the PI3K/AKT/NOS3 pathway in oxLDL-induced HUVECs. Collectively, these results demonstrated that hsa_circ_0004543 may play a vital role in the development of AS and affect the proliferation of HUVECs, providing a potential target for treating endothelial cell damage in AS.

## Introduction

Atherosclerosis (AS) is the main cause of coronary heart disease, cerebral infarction, and peripheral vascular disease (Pirillo et al., 2013; Herrington et al., 2016). Although various drugs have been developed to treat AS, the morbidity and mortality of this disease have declined significantly in developed countries, but not in some developing countries (Gao et al., 2020). AS is closely associated with lipid infiltration, endothelial cell injury, and vascular smooth muscle cell migration and proliferation. Oxidative modification and elevated levels of low-density lipoprotein are significant risk factors for AS (Pirillo et al., 2013; Di Pietro et al., 2016). Activated endothelial cells can be modified by oxidized low-density lipoprotein (oxLDL) and lead to AS (Pirillo et al., 2013).

Circular RNAs (circRNAs) are a novel type of non-coding RNA mainly composed of exons. To date, previous studies have shown that these are associated with a variety of human diseases, including malignant tumors, cardiovascular diseases, diabetes, and chronic inflammatory diseases (Stoll et al., 2018; Wang et al., 2018; Kristensen et al., 2019). It has previously been reported that circRNA_0077930 regulated senescence in vascular smooth muscle cells via hyperglycemia-stimulated vascular endothelial cell exosome production (Wang et al., 2020). Hsa_circ_0029589 may regulate proliferation and apoptosis of vascular smooth muscle cells stimulated by oxLDL (Yu et al., 2020). Moreover, inhibition of circDHCR24 alleviates smooth muscle cell proliferation and migration (Peng et al., 2020). Liu et al. (2020) summarized the functions and mechanisms of circRNAs in AS, which demonstrated that circRNA can regulate the proliferation, migration, and apoptosis of endothelial cells directly. In the process of AS, oxLDL activates endothelial cells and circRNA can combine with target genes or proteins to regulate the proliferation and migration of endothelial cells.

In the present study, differentially expressed circRNAs were identified in human umbilical vein endothelial cells (HUVECs). Their function was assessed with regard to cell proliferation and angiogenesis, and their mechanism of action involved the circRNA/miRNA/mRNA axis (Li et al., 2017). The data indicated that the expression levels of hsa_circ_0004543 were upregulated in HUVECs induced by oxLDL. Further functional experiments demonstrated its role in the proliferation and angiogenesis of HUVECs.

## Materials and Methods

### Cell Culture and Transfection

The present study was approved by the Ethics Committee of Minhang Hospital, Fudan University. HUVECs were obtained from the Chinese Academy of Sciences Cell Bank (Shanghai, China). These cells are usually used for angiogenesis experiments. Apoptosis of vascular endothelial cells (VECs) is a common event involved in the initiation of the pathogenesis of AS. The cells were cultured in RPMI-1640 medium in the presence of 10% FBS and at 37°C with 5% CO_2_. When HUVECs were in the logarithmic growth phase, they were treated with specific concentrations (0, 20, 40, 60, 80, and 100 mg/L) of oxLDL at specific time points (0, 0.5, 1, and 2 days). The expression levels of hsa_circ_0004543 were measured via qPCR. Small interfering (si)RNA sequences were used to knockdown hsa_circ_0004543 expression and si-normal control (si-NC) sequences were used as a control, which were purchased from Sangon Biotech (Shanghai, China). In order to construct the hsa_circ_0004543 overexpression plasmid, the hsa_circ_0004543 full-length complementary DNA (cDNA) was inserted into the pLCDH-CIR vector (BGI, Qingdao, China) and the normal vector was used as a control. Copanlisib is used as an inhibitor of the PI3K/AKT/NO3 pathway. According to the manufacturer’s instructions, Polyplus-transfection^®^ (Polyplus-transfection SA, Illkirch, France) was used to transfect HUVECs with the aforementioned RNA sequences at appropriate doses.

### RNA Extraction and RT-qPCR

The TRIzol^®^ reagent (Invitrogen, Thermo Fisher Scientific, Inc., Shanghai, China) was applied to extract total RNA from cells. cDNA was obtained from RNA using a cDNA synthesis kit (Takara Biotechnology Co., Ltd., Beijing, China). The SYBR Green PCR Kit (Takara Biotechnology Co., Ltd., Beijing, China) was used for qPCR. The primers and their sequences are listed in Table 1. The 2^–ΔΔ^ ^Ct^ method was used to analyze the relative expression of the genes examined.

**TABLE 1:** Primer sequences of the genes examined.

Hsa_circ0004543	F: ACCATCAGTGACCTGGACCTCTC
	R: ATCTCCTCAATGGCTGCCTTCT
PI3K	F: CCACGACCATCATCAGGTGAA
	R: CCTCACGGAGGCATTCTAAAGT
AKT	F: AGCGACGTGGCTATTGTGAAG
	R: GCCATCATTCTTGAGGAGGAAGT
eNOS	F: TGATGGCGAAGCGAGTGAAG
	R: ACTCATCCATACACAGGACCC
GAPDH	F: GGAGCGAGATCCCTCCAAAAT
	R: GGCTGTTGTCATACTTCTCATGG

### Cell Counting Kit-8 (CCK-8) Proliferation Assay

The CCK-8 (Dojindo Molecular Technologies, Inc., Kumamoto, Japan) was used to measure cell viability. In brief, HUVECs were cultured in 96-well plates with 5 × 10^4^ cells per well. Following incubation for 1–3 days, the microplate spectrophotometer was used to measure the absorbance at 450 nm.

### Flow Cytometry

The induction of apoptosis was detected by the Annexin V-FITC apoptosis detection kit (Invitrogen, Thermo Fisher Scientific, Inc., CA, United States). In short, HUVECs were stained with 1 μl of PI and 5 μl of Annexin V-FITC. The samples were incubated at room temperature in the dark for 20 min. The samples were analyzed by the ACCURI C6 flow cytometry software (BD Biosciences, CA, United States).

### Wound-Healing Assay

Following transfection, HUVECs (5 × 10^4^ cells/well) were incubated in the six-well plate and cultured until approximately 80% confluence. The cells were scraped and cultured for 1 day. Inverted microscopy was used to image the cells at 0 and 24 h.

### Transwell Assay

The cell migratory activity was measured using the Transwell assay (Corning, Inc., New York, United States). Briefly, 1 × 10^4^ cells were seeded in the upper chamber with free medium, while medium with 10% FBS was used in the lower chamber. Following culture at 5% CO_2_ with 37°C for 24 h, the cells were fixed with 70% ethanol and stained with 0.1% crystal violet. Images were captured under a microscope (Nikon Ti-s, Nikon Corporation, Shanghai, China), and the migrated cells were counted.

### Western Blot

The total protein was extracted from HUVECs using the ExKine cytoplasmic protein extraction kit (Abbkine Scientific, Co., Ltd., CA, United States) and quantified by the BCA protein analysis kit (Thermo Fisher Science, Inc.). The membrane containing 5% skimmed milk was sealed at room temperature for 60 min and incubated with the following antibodies: anti-PI3K, anti-AKT, anti-NOS3 (Univ, Shanghai, China), and β-actin (Univ, Shanghai China). The membrane was subsequently incubated with the secondary antibody and imaged.

### Statistical Analysis

The SPSS V23.0 and R (version 4.0.3) was used for statistical analysis. The difference in the mean between the groups was analyzed by the *t*-test. *p* < 0.05 was considered for significant differences.

## Results

### Examination of the Expression Levels of hsa_circ_0004543 in oxLDL-Induced HUVECs

**FIGURE 1:**
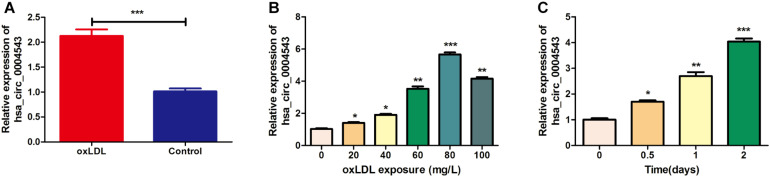
The expression of hsa_circ_0004543 is upregulated in HUVECs treated with oxLDL. **(A)** RT-qPCR analysis was used to detect hsa_circ_0004543 expression in HUVECs treated with oxLDL (80 mg/L). **(B)** Hsa_circ_0004543 expression in HUVECs was increased following treatment with oxLDL. **(C)** The relative expression of hsa_circ_0004543 in HUVECs treated with 80 mg/L oxLDL was increased with time. *p < 0.05, **p < 0.01, ***p < 0.001.

HUVECs were divided into two groups, those treated with oxLDL or without oxLDL, to compare the expression levels of hsa_circ_0004543. The results indicated that the expression levels of hsa_circ_0004543 in HUVECs treated with oxLDL (80 mg/L) were significantly higher than those without oxLDL (Figure 1A). In order to explore the dose–response relationship between oxLDL exposure and hsa_circ_0004543 expression, a series of oxLDL concentrations (0, 20, 40, 60, 80, and 100 mg/L) were used and the treatment effects were assessed at different time points (0.5, 1, and 2 days). The results indicated that the expression levels of hsa_circ_0004543 were increased following the increase in oxLDL concentration (Figure 1B). The highest levels were reached at 80 mg/L and were increased with the extension of the treatment duration (Figure 1C). Therefore, 80 mg/L oxLDL was incubated with HUVECs for 2 days in subsequent experiments.

### Knockdown of hsa_circ_0004543 Promotes the oxLDL-Induced HUVEC Proliferation

**FIGURE 2:**
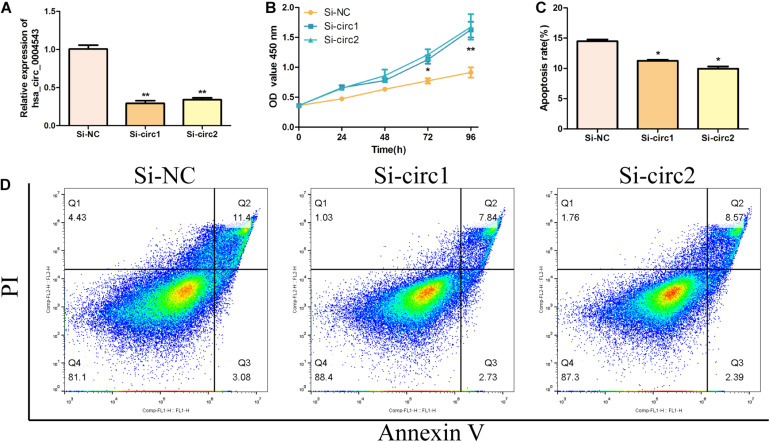
Silencing of hsa_circ_0004543 increases the proliferation of HUVECs induced by oxLDL. **(A)** The expression of hsa_circ_0004543 was suppressed by siRNAs. **(B)** The CCK-8 assay was used to measure the cell viability of oxLDL-induced HUVECs. **(C,D)** The apoptotic rate differences in HUVECs were assessed between si-NC and si-circ1&2. *p < 0.05, **p < 0.01.

To detect the biological function of hsa_circ_0004543 on HUVECs, siRNAs were used to inhibit hsa_circ_0004543 expression and examine its fold change. Surprisingly, both siRNAs designed could effectively suppress the expression of hsa_circ_0004543 (Figure 2A). The CCK-8 assay further demonstrated that hsa_circ_0004543 knockdown could promote oxLDL-induced HUVEC proliferation (Figure 2B). The flow cytometry assay indicated that the apoptotic rate of HUVECs transfected with si-NC was significantly higher than that in cells transfected with hsa_circ_0004543 (Figures 2C,D). In summary, the data indicated that the upregulated hsa_circ_0004543 expression was stimulated by oxLDL in HUVECs, whereas silencing of hsa_circ_0004543 could promote cell proliferation and reduce apoptosis.

### Hsa_circ_0004543 Knockdown Promotes Migration and Invasion of HUVECs

**FIGURE 3:**
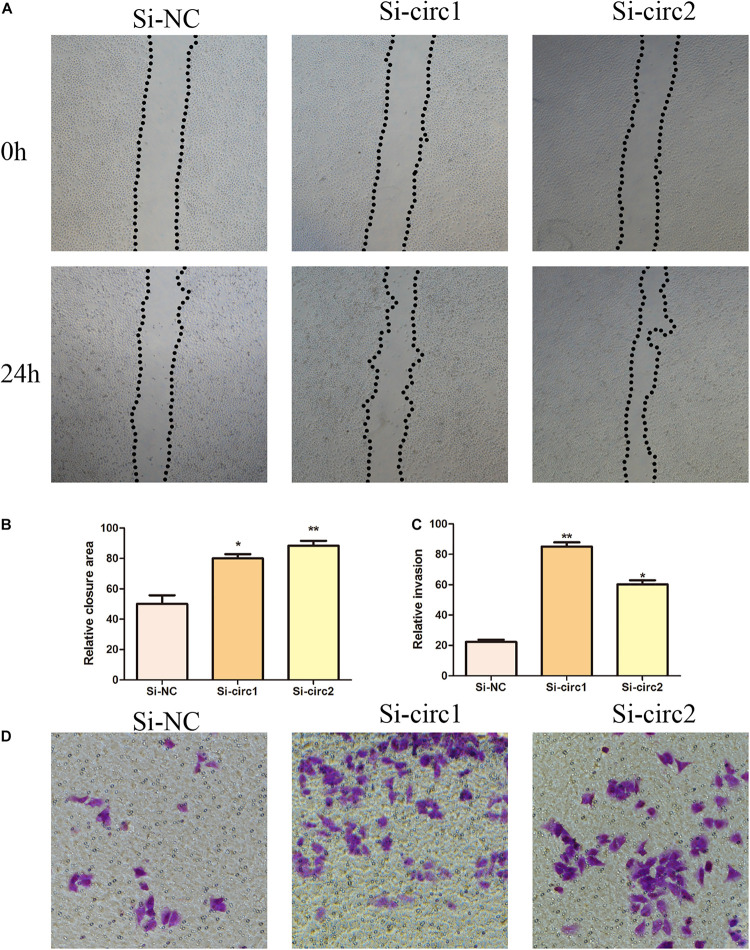
Effects of hsa_circ_0004543 knockdown on invasion and migration of HUVECs induced by oxLDL. **(A,B)** The migratory activity was detected at 0 and 24 h (black dotted line) by the wound-healing assay. **(C,D)** Cell invasion was evaluated by the Transwell assay (×100). *p < 0.05, **p < 0.01.

In order to elucidate the biological role of hsa_circ_0004543 in HUVECs induced by oxLDL, their angiogenic activity was assessed following transfection with si-circ1 and si-circ2. The wound-healing assay indicated that the control group exhibited significantly lower migratory activity than that of the oxLDL-induced HUVECs previously transfected with siRNAs (Figures 3A,B). In addition, the Transwell assay indicated that inhibition of hsa_circ_0004543 could enhance the invasive ability of HUVECs stimulated by oxLDL (Figures 3C,D).

### Silencing of hsa_circ_0004543 Activates the PI3K/AKT/NOS3 Pathway

**FIGURE 4:**
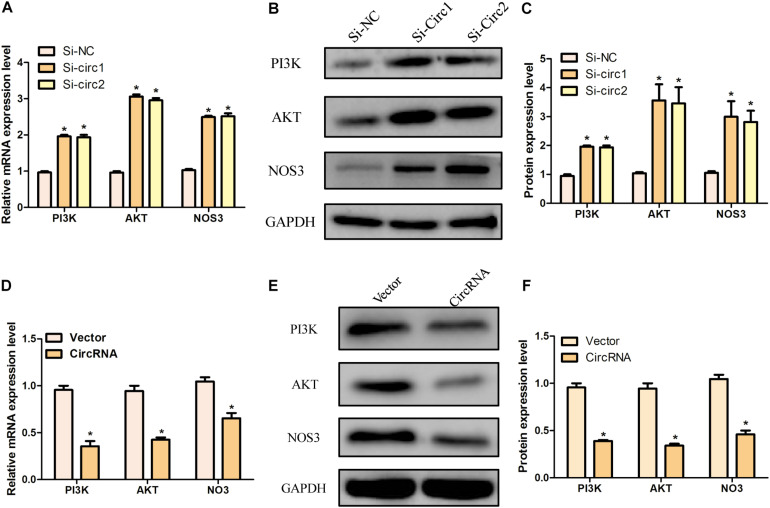
The knockdown and overexpression of hsa_circ_0004543 was influenced by the PI3K/AKT/eNOS pathway. **(A)** Relative mRNA expression of PI3K, AKT, and eNOS in oxLDL-induced HUVECs with silencing hsa_circ_0004543. **(B,C)** The expression of the proteins associated with the PI3K/AKT/eNOS pathway with silencing hsa_circ_0004543. **(D)** Relative mRNA expression of PI3K, AKT, and eNOS in oxLDL-induced HUVECs with overexpression hsa_circ_0004543. **(E,F)** The expression of the proteins associated with the PI3K/AKT/eNOS pathway with overexpression hsa_circ_0004543. *p < 0.05.

**FIGURE 5:**
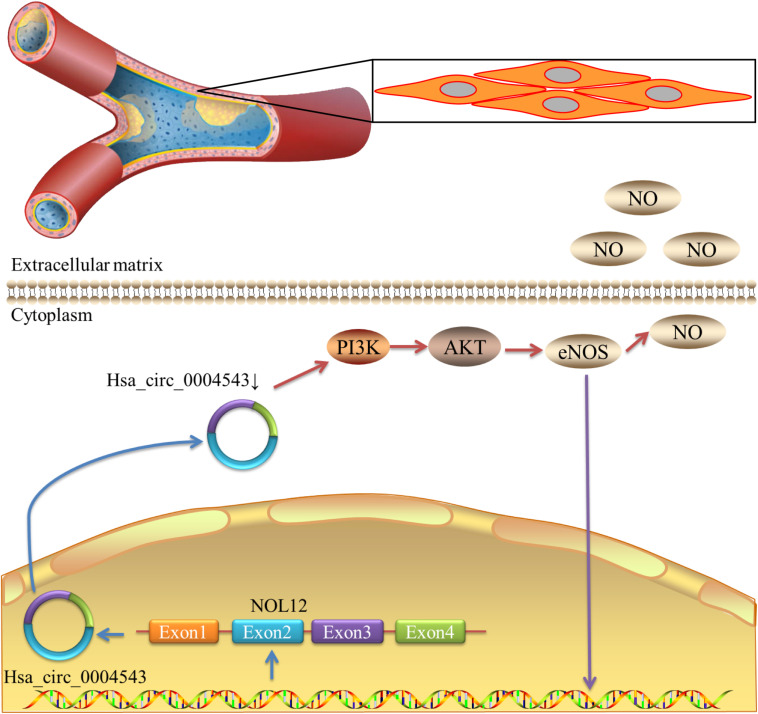
Schematic diagram demonstrating that the decrease in hsa_circ_0004543 expression activates the PI3K/AKT/eNOS pathway.

In order to further clarify the mechanism of hsa_circ_0004543 involved in cell invasion, the mRNA and protein expression levels of PI3K, AKT, and NOS3 were assessed. Knockdown of hsa_circ_0004543 led to a significant increase in the mRNA expression levels of PI3K, AKT, and NOS3 in HUVECs induced by oxidized low-density lipoprotein (Figure 4A). As shown in Figures 4B,C, the expression pattern of these three proteins was similar. It is interesting to note that PI3K, AKT, and NOS3 mRNA and protein expression levels were suppressed following overexpression of hsa_circ_0004543 (Figures 4D–F). These findings indicated that silencing of hsa_circ_0004543 could promote the migration of HUVECs and activate the PI3K/AKT/NOS3 pathway, which led to improvement of the angiogenic activity of endothelial cells (Figure 5).

## Discussion

VECs are the most vulnerable cells of the blood vessels. The destruction of the surface cells and the cellular dysfunction are considered to be the initial and crucial steps in the pathogenesis of AS. The function of circRNAs in VEC biology has been rarely reported. The present study examined the effects of hsa_circ_0004543 on HUVECs treated with oxLDL, which play a significant role in the initiation of AS. The data were consistent with the results of a previous study (Li et al., 2017), indicating that hsa_circ_0004543 expression was significantly increased in HUVECs treated with oxLDL. The results suggested that hsa_circ_0004543 may be involved in the occurrence and development of AS.

Cumulative evidence has shown that circRNAs can exert their biological functions by regulating gene transcription, translation, and expression of regulatory proteins, and by sponging various miRNAs, which are deregulated in human cardiovascular diseases (Lyu and Huang, 2017; Altesha et al., 2019; Fasolo et al., 2019). Previous studies that examined the function of circRNAs demonstrated that hsa_circ_0029589 regulated proliferation and apoptosis in oxLDL-induced HUVECs by regulating the miR-424-5p/IGF2 axis (Yu et al., 2020). The expression of circCHFR was upregulated in ox-LDL-induced VSMCs (Yang et al., 2019). Increased expression levels of hsa_circ_000595 were involved in the progression of aortic aneurysms by regulating apoptosis of HUVECs (Zheng et al., 2015). *In vitro* experiments demonstrated that silencing of RNAcZNF292 expression induced under hypoxic conditions could damage the germination and tubular formation and reduce the proliferation rate of HUVECs (Boeckel et al., 2015). In the present study, silencing of hsa_circ_0004543 promoted the proliferation, migration, and invasion of HUVECs induced by oxLDL, indicating that excessive hsa_circ_0004543 expression may be associated with disrupting VEC-injury repair.

The association between hsa_circ_0004543 and endothelial cell invasion was assessed by monitoring HUVEC invasion. NOS3 (also named eNOS) is an important downstream mediator of the PI3K/AKT pathway (Zhan et al., 2020). The PI3K enzyme is a kinase that catalyzes the phosphorylation of phosphoinositide and is widely expressed in various types of cells and participates in various biological processes. PI3K can be divided into the three following subtypes: type I, type II, and type III (Torrealba et al., 2018). AKT is also divided into three types, namely, kinase-α, -β, and -γ, respectively, which are significant downstream targets of PI3K. Following phosphorylation by PI3K, AKT acts on the downstream target proteins caspase-9, Bad, glycogen synthase kinase-3β, and eNOS. AKT is also involved in cell proliferation and apoptosis (Yoon, 2017; Zhao et al., 2017). Nitric oxide (NO) is not only an endogenous anti-atherosclerotic factor, but also an important indicator of endothelial cell function. If the concentration of NO decreases, it will lead to an increase in the concentration of free calcium ions in VECs. It can also alter cell permeability and promote VEC dysfunction and AS (Katakam et al., 2013). NO is mainly produced by endothelial cells following catalysis by eNOS. AKT is activated by PI3K and phosphorylates eNOS to promote the synthesis and release of endogenous NO, which protects VECs (Hao et al., 2017; Li et al., 2018).

circPIP5K1A can activate KRT80 and the PI3K/AKT pathway to promote gastric cancer by regulating miR-671-5p (Song et al., 2020). In ovarian cancer, circRHOBTB3 may act as an inhibitor and suppress tumorigenesis by inactivating the PI3K/AKT pathway (Yalan et al., 2020). Recent evidence has shown that the PI3K/AKT/eNOS axis also plays an important role in the progression and instability of atherosclerotic plaques. This occurs due to the increase of NO, the decrease in the concentration of free calcium, the change in cell permeability, and the induction of vasodilation (Nasirzadeh et al., 2019). In the present study, knockdown of hsa_circ_0004543 was associated with the activation of the PI3K/AKT/eNOS pathway in HUVECs induced by oxLDL. It was demonstrated that hsa_circ_0004543 may participate in angiogenesis by regulating the aforementioned pathway.

In summary, the present study demonstrated that the expression levels of hsa_circ_0004543 were upregulated in HUVECs induced by oxLDL. Its abnormal expression suppressed the proliferation, migration, invasion, and the activation of the PI3K/AKT/eNOS pathway in VECs. These findings improve the current understanding of the association between circRNAs and AS and support their application as therapeutic targets for the treatment of AS.

## Data Availability Statement

The original contributions presented in the study are included in the article/supplementary material, further inquiries can be directed to the corresponding author/s.

## Author Contributions

LH and WH contributed to the design of the present study. DL, YH, XZ, JL, XB, YL, PZ, MZ, and ZW performed the experiments. WH critically revised the manuscript. All authors drafted the manuscript.

## Conflict of Interest

The authors declare that the research was conducted in the absence of any commercial or financial relationships that could be construed as a potential conflict of interest.
